# Genetic polymorphisms of vascular endothelial growth factor (VEGF) associated with gastric cancer recurrence after curative resection with adjuvant chemotherapy

**DOI:** 10.1186/s12885-019-5702-5

**Published:** 2019-05-22

**Authors:** Yeon-Ji Kim, Woo Chul/ Chung, Kyong-Hwa Jun, Hyung-Min Chin

**Affiliations:** 1 Division of Gastroenterology, Department of Internal Medicine, St. Vincent’s Hospital,, The Catholic University of Korea, 93-6 Jungbu-daero, Paldal-gu, Suwon, Republic of Korea; 2 Department of Surgery, St. Vincent’s Hospital, The Catholic University of Korea, 93-6 Jungbu-daero, Paldal-gu, Suwon, Republic of Korea

**Keywords:** VEGF, Polymorphism, Gastric cancer, Prognosis

## Abstract

**Background:**

The relationship between polymorphisms in vascular endothelial growth factor (VEGF) and gastric cancer is still inconclusive. We investigated whether there is an association between VEGF genetic polymorphisms and risk of gastric cancer, and evaluated the recurrence of advanced gastric cancer after curative resection with adjuvant chemotherapy according to VEGF genetic polymorphisms.

**Methods:**

The association of functional single nucleotide polymorphisms (SNPs) of the *VEGF* gene (+936C > T, − 634G > C, − 2578C > A, + 1612G > A) were evaluated. Genotypes were determined by polymerase chain reaction-restriction fragment length polymorphism (PCR-RFLP) analysis. A total of 151 patients with gastric cancer were enrolled, and the control group consisted of 413 individuals with esophago-gastroduodenoscopy who were randomly selected through health screening. All of the enrolled patients had curative resections with completion of adjuvant capecitabine and oxaliplatin combination chemotherapy and the initial metastatic cases were excluded. During the regular follow-up protocol, the episodes of the recurrence were documented and the specific genotype and allelic frequencies were evaluated.

**Results:**

As for the cancer risk, there were no significant differences in specific genotypes and allelic frequencies. The mean follow-up period was 28.82 ± 30.92 (12 ~ 72) months and the recurrence rate was 28.3%. In the patients carrying the 936-C allele, the recurrence rate of gastric cancer was high (*P* = 0.02). Disease-free interval was significantly different between the patients carrying the 936-CC and 936-CT/TT genotype (*P* = 0.02).

**Conclusions:**

VEGF 936-C allele is associated with poor prognosis, but not risk of gastric cancer. In the patients carrying the 936-C allele, more potent adjuvant treatment would be considered.

## Background

Gastric cancer is the third and fifth-leading cause of cancer-related deaths in men and women, respectively worldwide [1]. Populations in eastern Asia have the highest gastric cancer burden in the world, and this region accounts for more than 60% of all gastric cancer cases [2]. The occurrence of gastric cancer involves several factors such as genetic, environmental and infectious agents, including *Helicobacter pylori*, having a cumulative effect in the early steps of gastric carcinogenesis [3].

Angiogenesis, the formation of new blood vessels from pre-existing vasculature is one of the hallmarks of cancer and plays a pivotal role in carcinogenesis by influencing growth, invasion, and metastasis [4]. Vascular endothelial growth factor (VEGF) is considered an important mediator of angiogenesis and an essential factor in cancer development and progression. VEGF promotes formation of new blood vessels, and subsequently enhances vascular permeability of endothelial cells, which are crucial events in cancer formation, invasion and metastasis [5, 6]. The VEGF gene is located on chromosome 6p21.3 and consists of 8 exons and 7 introns. It is highly polymorphic, with at least 30 functional single-nucleotide polymorphisms (SNPs) in the 5′-untranslated region (UTR), 3′- UTR, and promoter regions [7]. Particular focus has been on SNPs found in those in regulatory regions because they may alter VEGF expression levels [8]. High VEGF levels are associated with malignancies such as breast, lung, kidney, ovarian, colorectal and stomach cancer [9–15]. Moreover, these increased levels are associated with advanced stages and poor survival in several types of cancer. Hence, VEGF polymorphisms may influence cancer susceptibility.

Several potentially functional SNPs (−634G > C, − 7 C > T, − 1498 C > T, −1154G > A,-2578 C > A and -2489C > T in the promotor or in the 5′-untranslated region (UTR) and + 936C29 > T and + 1612G > A in the 3′ -UTR) have been revealed and might affect the expression of the *VEGF* gene [16]. It is likely that only a small number of these polymorphisms and haplotypes actually have a functional effect on VEGF translation [17]. Previous studies suggested that +936C > T (rs3025039), −634G > C (rs2010963), −2578C > A (rs699947), and + 1612G > A (rs10434) are common SNPS in the VEGF, and they are reported to have a role in VEGF protein synthesis [18]. Several studies revealed that 2578C/A, and 634G/C genotypes appear to be associated with a higher VEGF expression whereas the 936 T allele is correlated to lower VEGF expression [19, 20].

Although there are controversies possibly derived from cell-specific effects of VEGF SNPs in the solid tumors, several previous studies reported that VEGF polymorphisms contribute to the development and prognosis of cancers including gastrointestinal cancer as well as lung cancer, breast and urogenital cancer [21–29].

The role of VEGF SNPs in clinical outcomes in patients with gastric cancer was previously studied retrospectively, producing inconclusive results, as there was selection bias for enrollment and a lack of uniform criteria for primary end points [30–34]. To date, here is limited data correlating VEGF polymorphism and the prognosis of gastric cancers.

We aimed to clarify the potential association of four VEGF genetic polymorphisms (+936C > T, −634G > C, −2578C > A and + 1612G > A) with gastric cancer risk, and evaluated the recurrence of advanced gastric cancer according to VEGF genotypes.

## Methods

A total of 151 patients with gastric cancer who were operated on at St. Vincent Hospital from January 2010 to January 2013 were enrolled prospectively. The control group comprised 413 healthy individuals with esophago-gastroduodenoscopy who were randomly selected through health screening during the same period of time. Patients eligible for this study were identified preoperatively as having histological gastric adenocarcinoma. They were offered participation, and were required to provide informed consent. Written permission was obtained from all the participants and the study was approved by the Research Ethics Committee of St. Vincent Hospital Affiliated with the Catholic University of Korea (VC09TISI0005).

After curative operation, patients were subjected to adjuvant combination chemotherapy. We treated patients with 3-week cycles of chemotherapy (oral capecitabine 1000 mg/m^2^ twice daily from the evening of day 1 until the morning of day 15 plus intravenous oxaliplatin 130 mg/m^2^ on day 1 of each cycle). All patients were well hydrated and standard prophylactic medications were administered to reduce toxic effects. Treatment was continued for 6 months until toxic effects reached our endpoints.

After the adjuvant chemotherapy, a follow-up of all patients was carried out according to our standard protocol (every 3 months for at least 2 years, every 6 months for the following 3 years, and every 12 months after 5 years). The check-up items included physical examination, tumor marker examination, and computed tomographic scans. Endoscopic examination was carried out regularly (every 6 months for at least 2 years, every 12 months for the following years). Recurrent and mortality events were recorded, and disease-free survival (DFS) was calculated for the prognosis assessment.

### DNA extraction

The association of functional SNPs of the *VEGF* gene with gastric cancer development was evaluated in a case-control study of gastric cancer patients. Peripheral venous blood samples (3 mL) were acquired from all subjects in heparin vacutainers. Genomic DNA was obtained from the peripheral blood lymphocytes of study subjects using the Genomic DNA Extraction Kit (Bioneer Corp., Daejeon, Korea). Genomic DNA was isolated according to the manufacturer’s protocol. All samples were collected prior to treatment.

### Detection of VEGF gene polymorphism

The VEGF genotypes were selected from the previous study [8]. The four VEGF genotype polymorphisms (+936C > T (rs3025039), −634G > C (rs2010963), −2578C > A (rs699947), and + 1612G > A (rs10434)) were determined by polymerase chain reaction-restriction fragment length polymorphism (PCR-RFLP) methods with PCR primer pairs listed in Table 1 [35–37].

**Table 1:** Specific primers according to four VEGF polymorphisms

+ 936 C > T (rs3025039)	5′-AGG AAG AGG GAC TCT GCG CAG AGC-3′ (forward)
	5′-TAA ATG TAT GTA TGT GGG TGG GTG TGT CTA CAG G-3′ (reverse)
-634 G > C (rs2010963)	5′- ATT TAT TTT TGC TTG CCA TT − 3′(forward)
	5′- GTC TGT CTG TCT GTC CGT CA − 3′(reverse)
-2578 C > A (rs699947)	5′ - GGC CTT AGG ACA CCA TAC C − 3′(forward)
	5′- CAC AGC TTC TCC CCT ATC C − 3′(reverse)
+ 1612 G > A (rs10434)	5′- CAC ATG CTG CAC GCG CAT CTC A − 3′(forward)
	5′- ACC CCA GGA AGG GGA GCA GGA − 3′(reverse)

The PCR was carried out in a 30-μL reaction mix containing 100 ng DNA template, 1 unit of Taq DNA polymerase (iNtRON Biotechnology, Seoul, Korea), 0.2 mmol/L deoxyribonucleotide triphosphate (dNTP) (pH 8.3), 0.5 μM forward primer, 0.5 μM reverse primer, 1 × PCR buffer with 1.5 mM MgCl_2_ and 50 mmol/LKCl.

**Fig. 1:**
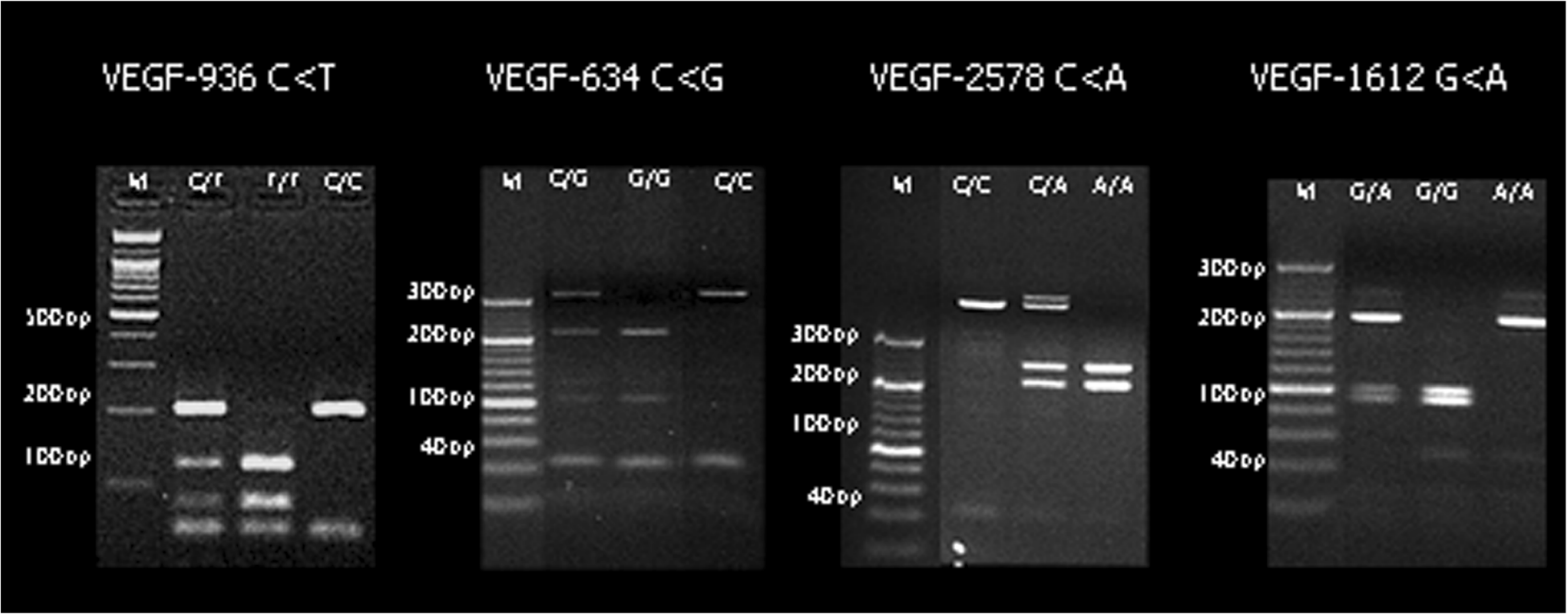
PCR for VEGF gene polymorphisms

The PCR reaction was carried out in a thermocycler and reaction conditions consisted of 95 °C denaturation for 5 min, 94 °C annealing for 30s, 62 °C annealing for 30 s, 72 °C annealing for 30 s (40 cycles) and 72 °C elongation for 10 min. The PCR products were digested with appropriate restriction enzymes (New England BioLabs, Beverly, MA, USA) at 37–65 °C for 2–16 h. The restriction enzymes were BstY1(2578C/A), MnlI (1612G/A), BsmFI (634C/G) and NlaIII (936C/G). The PCR and digestion products were analyzed with a 6% agarose gel with intercalating dye (ethidium bromide) staining. Selected PCR-amplified DNA samples were analyzed by DNA sequencing (Fig. 1).

### Statistical analysis

Unpaired t-test was used for continuous variable such as age. Chi-square test or Fisher’s exact test was for genotype frequency and recurrence risk or allele frequency and recurrence risk estimate. Correlation between incidence of cancer, and genotype was evaluated using the chi-squared test, if all cells of the contingency table contained at least 5 elements or Fischer’s exact test if the table included small cells. The odds ratio (OR) and confidence interval (CI) values of associations of genotype frequencies were calculated using univariate logistic regression, adjusting for potential confounding factor such as age, gender, smoking status and alcohol ingestion. Kaplan-Meier method was applied to plot survival curves and the statistical differences were analyzed by the log-rank test. All statistical analyses were two-sided, and *P* < 0.05 was considered statistically significant. Analyses were performed using SPSS version 19.0.

## Results

There were 94 males and 57 females in the gastric cancer group. The age of patients ranged from 29 to 83 years, with the average age of 66.16 ± 12.78 years. The baseline characteristics of gastric cancer patients and individuals of the control group are shown in Table 2.

**Table 2:** Baseline characteristics of the patients with gastric cancer and controls

	Gastric Cancer *N* = 151	Non-cancer Control *N* = 413	*P*-value
Sex (Male: Female)	94:57	250:163	0.71
Age	66.16 ± 12.78	51.45 ± 12.24	0.01*
Current smoker	45	152	0.12
Current alcohol drinker	48	160	0.13
Initial stage	IA (20) IB (37)		
	IIA (16) IIB (22)		
	IIIA (10) IIIB (14) IIIC (14)		
	IV (18)		

*value means that the *p*-value is significant

### VEGF polymorphism and risk of gastric cancer

The four VEGF genotype and allele distribution in the gastric cancer patients and the control subjects are presented in Table 3. All of the VEGF polymorphisms in the case and control patients were consistent with Hardy-Weinberg equilibrium. There were no significant differences in genotype subgroups and the frequencies between gastric cancer group and healthy controls.

**Table 3:** Comparison of genotype frequencies of the four polymorphisms according to the presence or absence of gastric cancer

VEGF SNP	Gastric Cancer *N* = 151 (%)			Non-cancer Control *N* = 413 (%)			*P*-value
−936 C > T	CC	CT	TT	CC	CT	TT	0.88
	98	48	5	258	141	14	
	(64.9)	(31.8)	(3.3)	(62.5)	(34.1)	(3.4)	
−634 C > G	CC	CG	GG	CC	CG	GG	0.80
	28	70	53	59	178	130	
	(18.5)	(46.4)	(35.1)	(14.4)	(43.1)	(31.5)	
−2578 C > A	CC	CA	AA	CC	CA	AA	0.15
	82	53	16	236	150	27	
	(54.3)	(35.1)	(10.6)	(57.1)	(36.3)	(6.6)	
−1612 G > A	GG	AG	AA	GG	GA	AA	0.35
	102	43	6	308	93	12	
	(67.5)	(28.5)	(4.0)	(74.6)	(22.5)	(2.9)	

### Recurrent gastric cancer after curative resection with adjuvant chemotherapy according to VEGF polymorphism

After curative resection, 16 patients were excluded from the study; there was no follow-up performed on 14 patients, one died a non-cancer-related death, and one experienced a fatal post-operative complication. A follow up of 28.82 ± 30.92 (12 ~ 72) months in a total of 135 patients with gastric cancer was performed (Table 4).

**Table 4:** Comparison of genotype frequencies of the four polymorphisms according to patients prognosis associated with metastasis and recurrence

VEGF SNP	Gastric cancer after curative surgery (*N* = 89)			Initial metastasis (*N* = 18)/ Recurrence after resection (*N* = 28)			*P*-value	
−936 C > T	CC	CT	TT	CC	CT	TT	0.30	
	57	30	2	35	10	1	.	44.37813
−634 C > G	CC	CG	GG	CC	CG	GG	0.91	
	19	45	25	9	25	12		
−2578 C > A	CC	CA	AA	CC	CA	AA	0.86	
	50	30	9	28	14	4		
−1612 G > A	GG	AG	AA	GG	GA	AA	0.72	
	64	21	4	30	14	2		

For the precise prediction of recurrence according to VEGF polymorphisms, we excluded very early gastric cancer (stage IA, 18 patients) and metastatic gastric cancer at the time of initial diagnosis (stage IV, 18 patients). Among remaining 99 patients (Table 5), the recurrence rate was 28.3% (28/ 99). Owing to the very small number of patients with the each polymorphism, the two genotypes were grouped together and analyzed dominant genetic model [rs3025039 CC and (CT + TT), rs2010963 GG and (CG + CC), rs699947 CC and (CA + AA] rs10434 GG and (AG + AA) in VEGF gene]. Compared with CT or TT genotype, 936-CC didn’t have statistical significance in increasing cancer recurrence (OR 0.37, 95% CI = 0.13–1.03, *P* = 0.06, adjust *P* = 0.9) (Table 6). The frequency of the 936-C allele was significantly higher in the recurrence group (*P* = 0.02, adjust *P* = 0.04) (Table 7).

**Table 5:** Baseline characteristics of patients with gastric cancer except stage IA and IV

	Gastric cancer after curative surgery *N* = 71	Recurrence after resection *N* = 28	*P-*value
Sex (Male: Female)	50: 21	22: 6	
Age	58.99 ± 11.92	62.89 ± 12.01	0.14
Current smoker	22	7	
Current alcohol drinker	20	10	
Chemotherapy	70	28	
Initial stage	IB (33)		
	IIA (8) IIB (14)	IIA (5) IIB (5)	
	IIIA (6) IIIB (5) IIIC (5)	IIIA (4) IIIB (7) IIIC (7)

**Table 6:** Comparison of genotype frequencies of the four polymorphisms according to gastric cancer recurrence after surgery

VEGF SNP		Advanced Gastric Cancer after curative resection (*N* = 71)	Recurrence after resection (*N* = 28)	*P*-value (adjust)	OR (95% CI)
−936 C > T				0.06 (0.09)	
Dominant CC vs CT + TT	CC	41	22		1.0
\	CT + TT	30	6		0.37 (0.13–1.03)
−634 C > G				0.75 (0.94)	
Dominant GG vs CG + CC	GG	23	10		1.0
	CG + GG	48	18		0.86 (0.34–2.16)
−2578 C > A				0.79 (0.97)	
Dominant CC vs CA + AA	CC	41	17		1.0
	CA + AA	30	11		0.88 (0.36–2.16)
−1612 G > A				0.46 (0.62)	
Dominant GG vs GA + AA	GG	51	18		1.0
	GA + AA	20	10		1.42 (0.56–3.59)

NOTE: Adjusted for age, gender, smoking status, and alcohol ingestion

*CI* confidence interval, *OR* odds ratio

**Table 7:** Comparison of allele frequencies of the four polymorphisms according to gastric cancer recurrence after surgery

VEGF SNP		Advanced Gastric Cancer after curative resection (*N* = 71)	Recurrence after resection (*N* = 28)	*P*-value (adjust)	OR (95% CI)
−936 C < T				0.02*	
				(0.04)	
	C allelic frequency	77.5%	89.3%		1.0
	T allelic frequency	22.5	10.7		0.41 (0.19–.91)
−634 C < G				0.21	
				(0.27)	
	C allelic frequency	44.4	35.7		1.0
	G allelic frequency	55.6	64.3		1.44 (0.81–2.53)
−2578 C < A				0.72	
				(0.84	
	C allelic frequency	74.6	76.8		1.0
	A allelic frequency	25.4	23.2		0.89 (0.46–1.69)
−1612 G < A				0.53	
				(0.66)	
	G allelic frequency	83.8	80.4		1.0
	A allelic frequency	16.2	19.6		1.26 (0.61–2.61)

* value means that the *p*-value is significant

*CI* confidence interval, *OR* odds ratio

### DFS after curative resection with adjuvant chemotherapy according to VEGF polymorphism

**Fig. 2:**
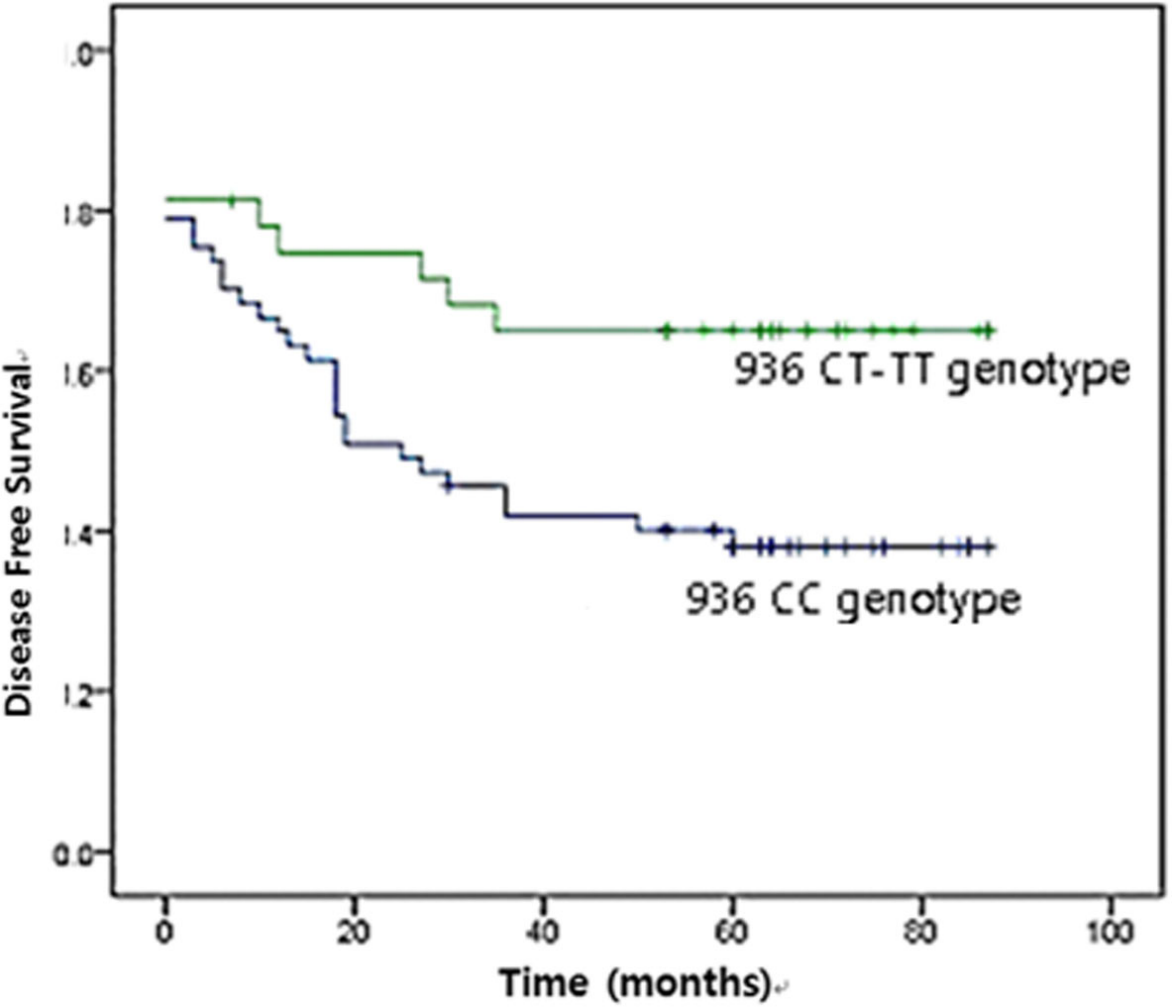
Kaplan-Meier curve according to VEGF 936-CC and 936-CT/−TT genotypes. (*P* = 0.02)

The proportion of 936-TT genotype was very small, we grouped 936-TT genotype and 936-CT genotype together and compared their DFS to that of the 936-CC genotype (dominant genetic model). In the patients with VEGF 936-CC genotype and 936-CT/−TT genotypes, DFS intervals were 51.13 ± 30.20 months and 62.26 ± 22.98 months, respectively. In a Kaplan-Meier survival curve, DFS of the patients with the VEGF 936-CC genotype was significantly lower than that of the others (*P* = 0.02) (Fig. 2).

## Discussion

Surgical resection with D2 lymphadenectomy has been established as standard procedure for non-metastatic advanced gastric cancer. The recurrence of gastric cancer is common, even though R0 resection was completed at the time of primary treatment. Survival rate depends on the stage of the cancer at the time of diagnosis; unfortunately survival rate after 5 years is below 30% in most patients with advanced gastric cancer. In clinical practice guidelines for gastric cancer treatment in Korea, adjuvant chemotherapy with either S-1 monotherapy or capecitabine and oxaliplatin combination therapy can be recommended after surgery for gastric cancer. Although the adoption of adjuvant chemotherapy after curative resection has been clear beneficial for recurrence and survival, new additional treatments are needed to increase the survival rate.

VEGF is a critical angiogenic factor that regulates capillary formation, vascular permeability, and endothelial cell proliferation, migration and differentiation. Several genotypes of VEGF polymorphisms influence VEGF production and are associated with susceptibility or severity of many diseases, including cancer. Previous studies suggested the association between VEGF polymorphism and risk of cancer, but the results remained inconclusive [30, 31, 38–40]. Particularly, in gastric cancer, several meta-analysis showed that there were debatable results [32–34, 41–46].

In the Chinese Han race and Japanese study, VEGF + 1612G > A gene polymorphism was found to be possibly associated with gastric cancer [34, 47]. A meta-analysis including these studies showed the 1612 A allele was a recessive gastric cancer susceptibility allele with a 60% increase of risk [37]. Recent meta-analysis showed + 1612 G/A G allele may decrease the gastric cancer risk in the Asian population [45, 46]. Some studies were examined for the relationship between cancer risk and − 2578 C > T and − 634G > C polymorphism [34, 48, 49]. In the Greek population, − 2578C > A and -634G > C polymorphism were associated with larger tumor size, poor differentiation, advanced stage of gastric cancer [48]. However, Chinese study didn’t revealed association these two SNP and the gastric cancer risk [49]. Meta-analyasis revealed that VEGF− 2578 C > A gene polymorphisms were found to be unassociated with gastric cancer risk, whereas the VEGF-634 G > C GG genotype was associated with gastric cancer risk [45].

In the Korean population, there is significant association of T allele-bearing genotypes with increased risk for stomach cancer development, which suggests that the VEGF + 936C > T polymorphism is a susceptibility [47]. In a meta-analysis, there was no association between VEGF + 936 C > T polymorphism and gastric cancer risk; 4 from Asian populations and 3 Caucasian populations [32]. In addition, a meta-analysis analysis of polymorphisms from only Asian populations showed that the VEGF *+*936C > T polymorphism is not associated with risk of overall cancer [44, 45]. In the present study, we investigated the association between four VEGF gene polymorphisms and gastric cancer risk and found that there were no significant differences in specific genotypes for gastric cancer risk.

Since VEGF has become a potential therapeutic target in the treatment of cancer, studies regarding its polymorphisms would be a basis for genetic tailored therapy in the near future [50]. As previously shown, the specific genotype of VEGF *+*936C > T might be an important determinant of VEGF plasma levels, which were closely relevant to lymph node metastasis [51, 52]. Although the polymorphism has been shown to affect the expression of the VEGF gene, the results remain inconclusive. Currently, there are a limited number of studies regarding VEGF gene polymorphisms and gastric cancer prognosis [30, 31, 53]. In a large-scale study, the VEGF 936-TT genotype was correlated with a worse overall survival compared with the VEGF 936-CC genotype [30]. However, more than 50% of patients had stage 0 or I early gastric cancers, and 8.4% had stage IV advanced gastric cancers. Only, 33.4% (167/500) of patients received adjuvant chemotherapy with different regimens. Therefore, a selection bias was present and may have resulted in an inaccurate conclusion. In another study, the VEGF − 634 G > C polymorphism was related to poor clinical outcome in advanced gastric cancer patients with chemotherapy [31]. However, more than 50% of patients (113/190) had stage IV advanced gastric cancer and only 41.6% (79/190) of patients received chemotherapy with FOLFOX. In the study on the risk for gastric cancer development and prognostic characteristics, there was a relation between the VEGF 936-TT genotype and poor prognosis. Stage I (18%) and stage IV (25%) patients were enrolled, and the primary end point was survival time without individual treatment protocol [53].

This study will help strengthen the knowledge on association of VEGF gene polymorphisms with gastric cancer prognosis for several reasons. Prospective designed study could eliminate a selection bias, and the exclusion of early mucosal cancer and initially metastatic cases gives a more refined study design. All patients underwent standard surgery with adjuvant chemotherapy and follow-up protocol according to clinical practice guidelines of Korea. In our results, the patients carrying VEGF 936-C allele showed higher recurrence rate of gastric cancer. Additionally, the VEGF 936-CC genotype was correlated with a lower DFS interval than the other genotypes.

Although the results on VEGF + 936 C > T polymorphism were ambiguous in gastric cancer, recently, similar results in other kind of cancers were reported. In a polymorphism study on non-small cell lung cancer, there was a correlation between the VEGF 936-TT genotype and lower risk of death compared to that with the CC genotype [54]. The VEGF 936-TT genotype played a protective role in the development of differentiated thyroid cancer [55].

This study had several limitations for the interpretation of results. First, the effect of gastric carcinogens such as *Helicobacter pylori* infection was not taken into consideration. Second, the relatively small sample size might not represent the entire general population. Furthermore, a relatively small number of patients carrying the VEGF 936 T allele would influence the results.

## Conclusions

In conclusion, our study suggests that the VEGF gene 936 + C > T polymorphism may be an independent prognostic marker in Korean gastric cancer patients. To improve the prognosis of gastric cancer, the analysis of VEGF gene polymorphisms may help identify a high-risk patient, and help clinicians and patients to seek active treatment.

## Abbreviations

CI: Confidence interval
DFS: Disease-free survival
dNTP: Deoxyribonucleotide triphosphate
OR: Odds ratio
PCR-RFLP: Polymerase chain reaction-restriction fragment length polymorphism
SNPs: Single nucleotide polymorphisms
UTR: Untranslated region
VEGF: Vascular Endothelial Growth Factor
